# JAK/STAT pathway: Extracellular signals, diseases, immunity, and therapeutic regimens

**DOI:** 10.3389/fbioe.2023.1110765

**Published:** 2023-02-23

**Authors:** Qian Hu, Qihui Bian, Dingchao Rong, Leiyun Wang, Jianan Song, Hsuan-Shun Huang, Jun Zeng, Jie Mei, Peng-Yuan Wang

**Affiliations:** ^1^ Department of Pharmacy, School of Medicine, Sir Run Run Shaw Hospital, Zhejiang University, Hangzhou, China; ^2^ Oujiang Laboratory, Key Laboratory of Alzheimer’s Disease of Zhejiang Province, Institute of Aging, Wenzhou Medical University, Wenzhou, China; ^3^ Department of Clinical Pharmacology, Xiangya Hospital, Central South University, Changsha, China; ^4^ Hunan Key Laboratory of Pharmacogenetics, Institute of Clinical Pharmacology, Central South University, Changsha, China; ^5^ Department of Orthopaedic Surgery, The Third Affiliated Hospital, Guangzhou Medical University, Guangzhou, China; ^6^ Department of Pharmacy, Wuhan First Hospital, Wuhan, China; ^7^ Department of Research, Center for Prevention and Therapy of Gynecological Cancers, Buddhist Tzu Chi General Hospital, Hualien, Taiwan; ^8^ Department of Thoracic Surgery, Xiangya Hospital, Central South University, Changsha, China

**Keywords:** JAK/STAT, disease progression, immune environment, mechanotransduction, therapeutic targets

## Abstract

Janus kinase/signal transduction and transcription activation (JAK/STAT) pathways were originally thought to be intracellular signaling pathways that mediate cytokine signals in mammals. Existing studies show that the JAK/STAT pathway regulates the downstream signaling of numerous membrane proteins such as such as G-protein-associated receptors, integrins and so on. Mounting evidence shows that the JAK/STAT pathways play an important role in human disease pathology and pharmacological mechanism. The JAK/STAT pathways are related to aspects of all aspects of the immune system function, such as fighting infection, maintaining immune tolerance, strengthening barrier function, and cancer prevention, which are all important factors involved in immune response. In addition, the JAK/STAT pathways play an important role in extracellular mechanistic signaling and might be an important mediator of mechanistic signals that influence disease progression, immune environment. Therefore, it is important to understand the mechanism of the JAK/STAT pathways, which provides ideas for us to design more drugs targeting diseases based on the JAK/STAT pathway. In this review, we discuss the role of the JAK/STAT pathway in mechanistic signaling, disease progression, immune environment, and therapeutic targets.

## 1 Introduction

Studies have shown that activation of the JAK/STAT pathway promotes the development and progression of various diseases, including various inflammatory diseases, lymphomas, leukemias, various solid tumors, and so on. Their relationship and mechanisms have become crucial for the treatment of various diseases. The JAK/STAT pathway is an important cascade of signal transduction for multiple growth factors and cytokines, which regulates gene expression and cell activation, proliferation, and differentiation (Reddy et al., 2019; Xin et al., 2020; Awasthi et al., 2021).

**FIGURE 1:**
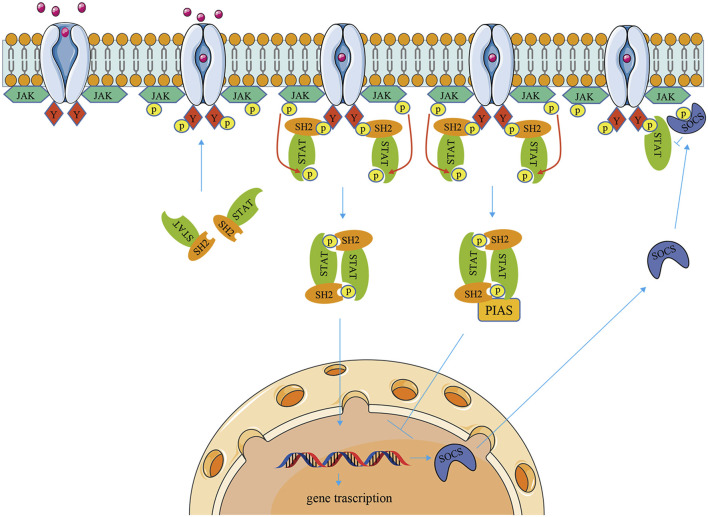
The signaling mechanism of the JAK/STAT pathway. When cytokines bind to transmembrane receptors, receptor-associated JAKs are activated, which then phosphorylate STAT proteins. Activation of STAT proteins forms homo- or heterodimers that are transferred to the nucleus and regulate gene transcription. The JAK/STAT pathway is negatively regulated through SOCS and PIAS.

The JAK/STAT pathway has three components: cellular receptors, JAK protein, and STAT protein. The JAK family is a group of non-transmembrane tyrosine kinases, which is mainly composed of four members: JAK1, JAK2, JAK3, and TYK2 with molecular weights ranging from 120 to 140 kDa. JAK1, JAK2, and TYK2 are ubiquitous, while JAK3 is mainly expressed in hematopoietic cells (Villarino et al., 2015; Banerjee et al., 2017). There are seven members of the STAT family in a mammal: STAT1, STAT2, STAT3, STAT4, STAT5A, STAT5B, and STAT6 (Banerjee et al., 2017). Each member of the STAT family can be activated by a variety of cytokines and associated JAKs (O'Shea et al., 2015). First, cytokines bind to the corresponding transmembrane receptors and induce dimerization then activating JAK kinases couple to and phosphorylate the receptors. Second, the tyrosine residues on the catalytic domain of the receptor are phosphorylated to form a docking site in which STAT proteins with SH2 domains are recruited to this docking site, and STATs are phosphorylated and form homodimers or heterodimers. Finally, dimerized STATs dissociate from receptors and translocate into the nucleus, where they bind to DNA-binding sites and regulate gene transcription (Murray, 2007; Muller, 2019). Therefore, the activation of the JAK/STAT signaling cascade pathway is necessarily influenced by upstream extracellular cytokines and downstream JAK/STAT family protein types. For example, IFN-α/β activate STAT1, STAT2, and STAT4 via JAK1 and TYK2, whereas IFN-γ actives STAT1 or STAT5 via JAK1 and JAK2. IL-6 and IL-11 activate STAT1, STAT3 via JAK1, JAK2, and TYK2, but IL-12 and IL-23 activate STAT3 and STAT4 via JAK2 and TYK2. At the same time, STAT can be directly activated independently of JAK pathways, such as epidermal growth factor (EGF), platelet-derived growth factor (PDGF), and mitogen-activated protein kinase (MAPK). In addition, the JAK/STAT pathway receives regulation by multiple mechanisms, including that PIAS inhibits gene transcription by directly binding to STAT dimers and thereby blocking STAT binding to DNA. And SOCS protein can negatively regulate the JAK/STAT signaling cascade by inhibiting JAK activity, competing with STAT to bind phosphorylation sites on cytokine receptors, and inducing STAT proteasomal degradation (Boyle et al., 2009; La Manna et al., 2021) (Figure 1).

## 2 JAK/STAT pathway in disease progression

**FIGURE 2:**
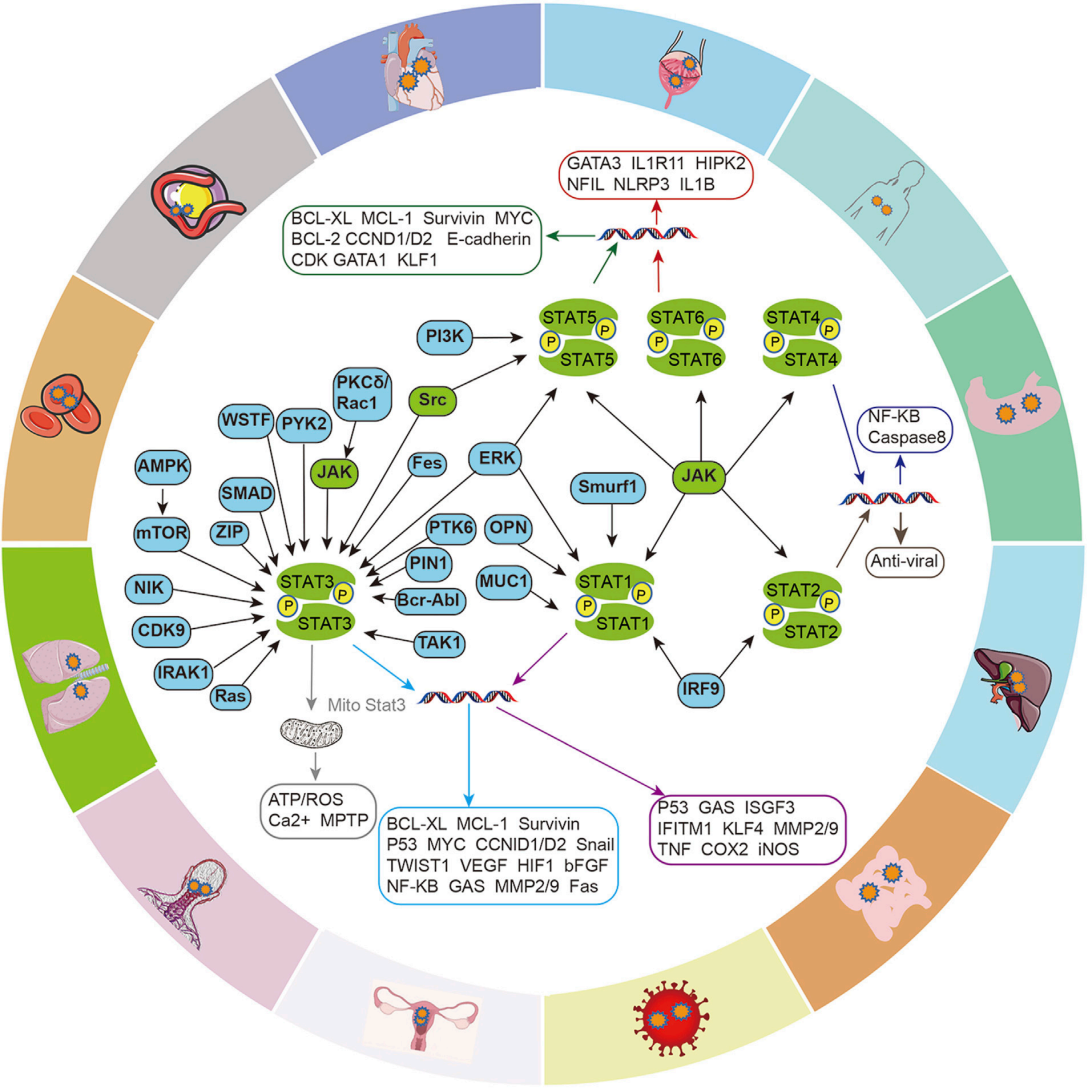
Intracellular signaling crosstalk of the JAK/STAT pathway. Different signaling proteins activate different STATs and induce the transcription and expression of genes for different cellular functions, including cell cycle, apoptosis, cell proliferation, epithelial-mesenchymal transition (EMT), angiogenesis, inflammatory factor production, etc. which in turn are involved in the development of various diseases.

The JAK/STAT signaling axis is a central pathway that mediates the cellular inflammation response, and carcinogenesis, and participates in the transduction of cellular physiological signals, such as renin-angiotensin signaling, insulin-like growth factor (IGF-IR) signaling (Figure 2). STAT promotes the transcriptional activation of target genes in response to specific extracellular stimuli (including cytokines, growth factors, and other agents) through tyrosine phosphorylation-mediated activation, most of which is mediated by JAKs, but with the interaction of multiple intracellular signaling proteins, then affecting key cellular processes, including differentiation, proliferation, survival and functional activation, which in turn are involved in the development of various diseases.

### 2.1 JAK/STAT pathway in oncopathology

In oncology research, JAK/STAT is attracting more and more attention, increasingly studies show that STAT3 is constitutive activated in tumors and involved in cellular carcinogenesis (Teng et al., 2014). The pathway is involved in a variety of malignant tumors, including leukemia (Dufva et al., 2018), multiple myeloma (Hu and Hu, 2018), lymphoma (von Hoff et al., 2019), head and neck cancer (Sen et al., 2015), colon cancer (Neradugomma et al., 2014), gastric cancer (Mimura et al., 2018), hepatocellular carcinoma (Ren et al., 2019), pancreatic cancer (Chen et al., 2018), breast cancer (Shao et al., 2021), melanoma (Guenterberg et al., 2010), ovarian cancer (Bagratuni et al., 2020), lung cancer (Patel et al., 2019) and prostate cancer (Kroon et al., 2013).

Cancer stem cells (CSCs), a subpopulation of tumor cells with stem cell properties that self-renew and give rise to a variety of more differentiated cells, are a key driver of tumor progression (Dean et al., 2005; Dorritie et al., 2014; Avgustinova and Benitah, 2016). Studies have shown that cancer stem cells have been proposed to explain the development of cancer and resistance to treatment, and activation of the JAK/STAT signalling pathway, or induction of other signals that interact with the JAK/STAT pathway, can promote the production and acquisition of drug resistance by cancer stem cells (Shibue and Weinberg, 2017; Dongre and Weinberg, 2019). Researches show that STAT3 is critical for tumor transformation downstream of oncogenes Src and Ras. Src induces tyrosine phosphorylation and transcriptional activity of STAT3, and Ras phosphorylates STAT3 at Serine 727, which is required for localization to mitochondria. In turn, mitochondrial STAT3 supports Ras oncogenic transformation by supporting a metabolic shift (Avalle et al., 2012). Cao et al. found that STAT3 was consistently activated in Src-transformed cell lines, and the interruption of the STAT3 signal blocked the transformation of mouse fibroblasts by Src oncoprotein (Cao et al., 1996). Furthermore, it has been reported that oncogenes such as Bcr-Abl, v-Eyk, v-Ros, and v-Fps may play similar functions (Mankan and Greten, 2011). JAK/STAT pathways are also involved in various aspects of tumor development, such as invasion and metastasis (Loh et al., 2019). For example, abnormal activation of IL-6-mediated JAK/STAT3 signal transduction frequently occurs in human cancers and is involved in transformation, tumorigenicity, EMT, and metastasis. IL-6/JAK2/STAT3 activation induces EMT by up-regulating EMT-induced transcription factors (EMT-TFs, Snail, Zeb1, JUNB, and Twist-1), and enhances cell motility by activating focal adhesion kinase (FAK), which enhances metastasis (Jin, 2020). Xiao et al. demonstrated that IL-6 can promote EMT in peritoneal mesothelial cells, which is related to the activation of the JAK/STAT pathway (Xiao et al., 2017). Furthermore, reviews have concluded the effects of JAK/STAT3 activation on EMT by multiple intracellular signals protein, including PTK6, Williamʹs syndrome transcription factor (WSTF), Pin1, PYK2, SMAD4, RAC1, and other signals protein (Jin, 2020). NF-κB signaling has been identified as a major pathway to induce inflammation in tumors, where STAT3 directly interact with NF-κB family members to capture it in the nucleus, thereby promoting constitutive activation of NF-κB (Lee et al., 2009), leading to many oncogenic and inflammatory genes activations (Lee et al., 2009; Yu et al., 2009). Ruan et al. found that overexpression of OCT4 (a marker for cancer stem cells in ovarian cancer) increased the activation of the JAK/STAT pathway, especially JAK1 and STAT6, and promoted the translocation of STAT6 from the cytoplasm to nuclear in non-SP cells (CSC-like side population cells), thereby increasing the expression of Cyclin D1, c-Myc, and Bcl-2 (Gough et al., 2013; Ruan et al., 2019). As mentioned earlier, JAK/STAT can be activated by a variety of cytokines, thereby transducing and activating a variety of downstream signaling pathways in cells (Figure 2).

Activated JAKs also induces the activation of other downstream signaling cascades, including the MAPK and PI3K/AKT pathways. Studies demonstrate that ERK signaling regulates MHC II expression in spinal cord microglia through regulation of the STAT1 phosphorylation and promotes bone cancer pain (Song et al., 2017). AMPK inhibits tumor proliferation by suppressing STAT3 activation (Lee et al., 2011). Furthermore, STAT5 was found to form a complex with ERK1/ERK2 in colorectal cancer cells, suggesting a cross-talk between STAT5 and MAPK signaling pathways in the development of human colorectal cancer (Xiong et al., 2009). However, another recent study showed the presence of STAT5 in PI3K immunoprecipitation in leukemic bone marrow cells (Harir et al., 2007), but no STAT5-PI3K complexes were found in CRC cells. The specific cell type and tumor microenvironment may explain this phenomenon. It has been reported that STAT3 down-regulates the expression of important proteins related to apoptosis induction, including P53 (Niu et al., 2005), IFN-β (Wang T. et al., 2004), Fas and its ligands, and BAX (Kunigal et al., 2009; Liang et al., 2011). Abnormal activation of STAT3 also leads to abnormal overexpression of various proteins, including Mcl-1, Bcl-2, Bcl-xl, survivin, Cyclin D1, c-Myc, and VEGF, which leads to tumor development (Gao et al., 2005; Verma et al., 2010). In Barbara’s review, he mentioned that STAT was related to autophagy. PKR-eIF2A pathway is an important inducer of autophagy, and STAT3 inactivates this pathway through binding to PKR and inactivation of eIF2A phosphorylation (Jonchere et al., 2013; Bagca et al., 2016). In addition to STAT3, constitutive activation of STAT1 and STAT5 was also shown in tumor cells and tumor tissues. In chronic myeloid leukemia (CML) and myeloproliferative diseases induced by TEL-JAK2, STAT5 is activated by a variety of hematopoietic and non-hematopoietic cytokines and growth factors to promote the development of these tumors (Lin et al., 2000; Levis et al., 2002; Subramaniam et al., 2020). However, activation of STAT1 usually appears to promote tumor cell apoptosis and anti-proliferative effects. Tumors are more likely to develop in STAT1-deficient mice (Shankaran et al., 2001; Avalle et al., 2012). However, some studies have also shown that STAT1 can induce platinum resistance in breast cancer, which may be independent of the activation of JAK2/3 (Stronach et al., 2011).

In summary, the JAK/STAT pathway is involved in the activation and transduction of various signaling pathways related to tumorigenesis and development, suggesting that the JAK/STAT pathway may be another new target for cancer treatment.

### 2.2 JAK/STAT pathway in other diseases

Recent studies have shown the involvement of JAK/STAT in multiple diseases and their physiological processes. For example, studies have found that RA phosphorylates JAK2 by binding to AT1, thereby activating JAK and STAT signaling pathways to mediate VCSM growth, migration, and remodeling (Mehta and Griendling, 2007). IGF-IR exerts signaling effects by activating the JAK/STAT pathway. A study unveiled that miR-326 targets MDK to regulate the progression of cardiac hypertrophy by blocking JAK/STAT and MAPK signaling pathways (Zhang J. et al., 2020). Melatonin may have protective and therapeutic effects on hypercholesterolemia by regulating vaspin, STAT-3, DDAH, and ADMA signaling pathways (Sezgin et al., 2020). Increased levels of STAT-1 promote SMC (Smooth muscle cell) de-differentiation, whereas high levels of STAT-3 drive SMC into a more mature phenotype (Kirchmer et al., 2014). Therefore, the study of the JAK/STAT pathway can help us gain a deeper understanding of the pathological and pharmacological mechanisms of multiple diseases.

## 3 JAK/STAT signaling regulation of the immune environment

The role of the JAK/STAT pathway is critical in immune regulation which has attracted increasing attention. STAT transcription factors were regulated by many cytokines, so as to control the immune response and induce tumor immune escape, promote or inhibit the expansion and activation of various immune cells (Table 1).

**TABLE 1:** JAK/STAT pathway mediates the effect of cytokines on immune cells.

**Cytokine**	**STAT**	**Effect**	**PMID**
IFN-γ	STAT1 deficiency	reduce suppression by MO-MDSCs	18272812
IFN-α	STAT1	induce HSC proliferation and differentiates into CDP	19212321
IFN-α	STAT1	regulators of IL-12 production by DCs	16618773
IFN-α/β	STAT1	maintain accumulation of proliferative NK cell	12370359
IL-12/IFN-γ	STAT1	enhance T-cell infiltration and tumor growth inhibition	17634555
IL-12	STAT1 deficiency	increase tumor-specific CTL activity	16618773
		increases the CD8 T-cell density	
IFN-α	STAT2	antagonize stress-dependent expansions of T cells	11163195
iNOS/VEGF	STAT3	increase MDSC suppressive function	22529296
G-CSF	STAT3	promotes the development of MDSCs	25649351
Flt3L	STAT3	increase MDSC suppressive function	24639346
GM-CSF	STAT3	MDSCs expand and suppress antitumor immunity	27199222
IL-6/IL-10/VEGF	activate STAT3	enhance the number of MDSC	25238263
			29100353
			22529296
IL-6	STAT3	increase CD11b^+^CD14^+^HLA-DR- myeloid cells	25238263
IL-6	STAT3	suppress DC maturation	15356132
VEGF/IL-10	STAT3	inhibit functional DC maturation	16288283
			14702630
			16371463
			14688356
			14702634
Flt3L	STAT3	stimulate pDC generation	20933441
			14670306
IL-10/IL-21	STAT3	NK-cell activity impaired	24891320
IL-6/IL-10/VEGF/HGF	STAT3	regulating the activity of NK cells, toxicity function and interaction with other immune system components	16288283
			27148255
APT2	STAT3	promotes Th17 cell differentiation	33029007
IL-10/TGF-β	STAT3	induction of the Treg phenotype of the transformed CD4^+^ T cells	16766651
IL-6	STAT3	promoting naïve CD4^+^ T cell differentiation into inflammatory Th17 cells	26912317
IL-6	STAT3	inhibit the differentiation of Th9 cells	26976954
IL-6	STAT3	mediated Th17 differentiation	19564351
			16688182
IL-6	STAT3	maintains the mitochondrial membrane potential during CD4 cell activation	25974216
			34809691
IL-2	STAT3	induce CD4^+^CD25^+^ Tregs	16645171
			14500638
			15611254
IL-10/TGF-β	STAT3	tumor-derived CD4^+^CD25^+^ regulatory T cells suppress DC maturation	16612596
IL-17A/IL-6/IL-23	STAT3	modulating the balance of Th17 and Treg cells, as well as in promoting CD4 T cell proliferation	20493732
IL-10	STAT3	deactivation of macrophages and neutrophils	10023769
IL-10	STAT3	promote the formation of M2 macrophages	23169551
IL-6/IL-10	STAT3	poor cytotoxicity and anti-tumor immune response	29222039
IL-12	STAT4	promote Th1 cells differentiation	11086031
T-bet	STAT4	Tfh cell production of IFN-γ	29212666
GM-CSF	STAT5	block pDC development	18342552
GM-CSF	STAT5	promotes CD103^+^ DC development	23033267
IL-2/IL-15	STAT5	accumulation of NK	28916644
			29105654
IL-2	STAT5	induce CD4^+^CD25^+^ Tregs	16645171
			14500638
			15611254
IL-10/TGF-β	STAT5	tumor-derived CD4^+^CD25^+^ regulatory T cells suppress DC maturation	16612596
IL-2 receptor beta	STAT5	regulate FoxP3 expression, and promote Treg differentiation	17182565
GM-CSF	STAT5	drug resistant to sunitinib	20406969
IL-4/IL-13	STAT6	activation of MDS, increases the suppressive function of MDSCs	19197294
			19197294
IL-4	STAT6	promote differentiation of Th2 cells	11086031
			8624821
IL-4	STAT6	restrict CD8^+^ T cell expression	18566374
IL-4	pSTAT6	alternative macrophage polarization	29343442
STING	STAT6	antiviral innate immunity	22000020

### 3.1 MDSC immunosuppressive function

As we know, Myeloid-derived suppressor cells (MDSCs) are immature myeloid cells and have immunosuppressive properties for adaptive immunity and innate immunity. MDSCs are derived from hematopoietic stem cells in bone marrow (Millrud et al., 2017). Signals from tumors and inflammatory tissues stimulate the differentiation of IMC (immature myeloid cells, the progenitors of MDSCs) to MDSC through the STAT pathway and promote their expansion (Salminen et al., 2019). Kim’s review has elucidated that VEGF, G-CSF, GM-CSF, Flt3L, and other anti-inflammatory cytokines (IL-4, IL-6, IL-10) can activate STAT signaling and thus regulate MDSC proliferation and activation (Ko and Kim, 2016). IL-6, IL-10, and VEGF can activate STAT3 on MDSC, which can enhance the number of MDSC (Jayaraman et al., 2012; Chen et al., 2014; Wu et al., 2017). MDSCs immunosuppressive mechanisms may be related to the expression of arginase-I (Vasquez-Dunddel et al., 2013), IDO (Yu et al., 2014), iNOS (Wu et al., 2017), and PD-L1 (Thorn et al., 2016) by JAK-STAT3 signals activation. STAT1 is a major transcription factor for IFN-γ mediated signaling activation and is involved in the upregulation of arginase 1 and iNOS expression by MDSCs (Kusmartsev and Gabrilovich, 2005). For example, research has shown that blocking IFN-γ or disrupting STAT1 partially impaired suppression by MO-MDSCs (Movahedi et al., 2008). Activation of STAT6 by IL-4, and IL-13 leads to activation of MDSC, which causes upregulation of arginase 1, inducible iNOS, and production of transforming growth factor-β (TGFβ), which then increases the suppressive function of MDSCs (Gabrilovich and Nagaraj, 2009).

### 3.2 DC development

Dendritic cells (DC) are discrete cell populations derived from hematopoietic stem cells (HSCs) and have important functions in immune surveillance (Gardner et al., 2020). DCs are produced by hematopoietic progenitor cells (such as CDP) under the control of exogenous cytokine signals and intrinsic transcriptional regulators (Collin and Ginhoux, 2019). The main cytokines involved in DC development include Flt3L, GM-CSF, and IFN-α, which respectively stimulate STAT3, STAT5, and STAT1, and each STAT has a different role in DC production (Yu et al., 2007). Studies have shown that STAT3 activation is an important factor for Flt3L to regulate DC development, and the absence of STAT3 in hematopoietic cells eliminates the effect of Flt3L on DC (Laouar et al., 2003; Sathaliyawala et al., 2010). Under steady-state conditions, GM-CSF regulates the generation of CD103^+^DC by inducing STAT5-ld2 signal activation (Li et al., 2012). STAT5 is considered to be the major GM-CSF response signal protein (Gouilleux et al., 1995). Studies have shown that GM-CSF used STAT5 to prevent the development of Flt3L-dependent pDC from the lineage-negative Flt3^+^ (lin^−^ Flt3^+^) bone-marrow subset (Esashi et al., 2008). STAT1-mediated IFNα promotes the proliferation of HSCs and then differentiates into CDP (Essers et al., 2009). IL-6 is the main cytokine controlling DCs differentiation *in vivo*. IL-6 mediates the inhibitory effect of bone marrow-derived DC maturation by regulating STAT3 phosphorylation in DC cells (Park et al., 2004). The inhibitory effect of STAT3 on DC maturation may also be caused by VEGF and IL-10 signaling (Wang T. et al., 2004). The functions of DC, T cells, natural killer (NK) cells, and neutrophils are significantly enhanced in tumor-bearing mice with Stat3^−/−^ hematopoietic cells (Kortylewski et al., 2005).

### 3.3 NK cell function

Natural killer (NK) cells are an important early effector in the innate immune system to resist multiple viral infections and eliminate tumor cells (Cooper et al., 2001). IL-15 is required for the maturation of NK cells at all stages of development (Becknell and Caligiuri, 2005). In Nguyen’s experiment, they found that in a virus-infected mouse model, the maintenance of IFNα/β on the accumulation of proliferative NK cells is mainly dependent on the production of IL-15 induced by STAT1 (Nguyen et al., 2002). In mice deficient in STAT1, T-bet, or MHC type I molecules, the maturation state of peripheral NK cells is impaired (Robbins et al., 2005), and they are more sensitive to viral and bacterial infections (Sugawara et al., 2004). STAT3 can also indirectly damage the function of NK cells by regulating the expression of NK cell activation receptor ligands and immune checkpoint proteins, such as NKG2D ligand MICA, PD-L1 (Rocca et al., 2013; Zhu et al., 2014; Cacalano, 2016). In a mouse model, STAT3-deficient NK cells enhance tumor immune surveillance and increase DNAM-1 and the lytic enzymes perforin and granzyme B secretion (Gotthardt et al., 2014). STAT5a and STAT5b are important transcription factors for the activation, proliferation, and maturation of NK cells in humans and mice (Vargas-Hernandez and Forbes, 2019; Kee et al., 2020). STAT5b-deficient NK cells have decreasing proliferation and toxicity after stimulation by IL-2 and IL-15 (Imada et al., 1998; Villarino et al., 2017), and circulating NK cells in STAT5b-deficient patients are also significantly reduced, resulting in low cytotoxicity (Bernasconi et al., 2006).

### 3.4 T cells

T cells are derived from lymphatic stem cells in the thymus. They are the most numerous and complex type of cells in lymphocytes that produce cellular immunity. IL-6 and IL-10 cytokines activate STAT3 on T cells, which is usually related to poor cytotoxicity and anti-tumor immune response (Wang et al., 2018). Studies have shown that IL-6 mediates the differentiation of naive CD4^+^ T cells into inflammatory Th17 cells through STAT3, while IL-10 targeting IL-10Rα has similar effects (Jones et al., 2016). Furthermore, studies showed that IL-6 maintains the mitochondrial membrane potential during CD4^+^ cell activation in a STAT3-dependent manner, thereby increasing mitochondrial Ca^+^ levels and promoting cytokine expression (Yang et al., 2015; Awasthi et al., 2021). Studies found that STAT3 bound to multiple genes involved in Th17 cell differentiation by using chromatin immunoprecipitation and massive parallel sequencing (ChIP-Seq) (Durant et al., 2010). STAT3 and FoxP3 can be used as transcription factors to regulate the biological function of Treg (Woods et al., 2018). IL-12 induces a high intensity of tumor-specific CTL activity in STAT1-deficient mice, increases the CD8^+^ T-cell density, and induces a T-cell-dependent tumor regression (Torrero et al., 2006). IL-2-induced FOXP3 expression in human Treg cells is mediated by STAT signaling, including both STAT3 and STAT5. STAT5 also can combine with the FoxP3 gene promoter, regulate FoxP3 expression, and promote Treg differentiation (Burchill et al., 2007). The phosphorylation of STAT3 by IL-6 will inhibit the Th9 cell differentiation, which was mediated by the suppression of IL-2 production and STAT5 signaling (Olson et al., 2016). IL-2 selectively up-regulated the expression of FOXP3 in purified CD4^+^CD25^+^ T cells which involved the binding of STAT3 and STAT5 proteins (Antov et al., 2003; Anderson et al., 2005; Zorn et al., 2006). And present data demonstrate that CD4^+^CD25^+^FoxP3^+^ regulatory T cells impede dendritic cell function which requires TGF-beta and IL-10 by activating STAT3 (Larmonier et al., 2007). Furthermore, IL-12 is able to transcriptionally regulate STAT4 and thus participated in the development and differentiation of Th1 cells and STAT6-deficient T lymphocytes failed to differentiate into Th2 cells despite under IL-4 stimulation (Ostrand-Rosenberg et al., 2000). Activation of the IFNγ/STAT1/IRF1 axis favors processing and the presentation of tumor antigens, in association with MHC class I or class II molecules (Avalle et al., 2012). Research has shown that endogenously secreted IFNs served to antagonize stress-dependent expansions of T cells through a STAT2-dependent pathway (Park et al., 2000). In addition, STAT1 hyper-phosphorylation can lead to impaired IL-23 signaling, which can in turn result in defective Th17 T cell responses (Smeekens et al., 2011).

Members of the STAT protein family are involved in regulating immune responses in the tumor microenvironment, including pro-tumor or anti-tumor inflammatory responses. On the one hand, abnormal STAT3 expression in tumors is correlated with MDSC and Th17 levels, affecting DCs and thus affecting anti-tumor response (Yu et al., 2009). IL-6 induces STAT3-mediated Th17 differentiation, which maintains inflammatory responses by releasing IL-17 and IL-23 and causes the secretion of VEGF and TGF in fibroblasts and endothelial cells (Langowski et al., 2006; Wang L. et al., 2009). STAT3-mediated IL-23 production also inhibited the proliferation of effector T cells (Kortylewski et al., 2009). STAT3 also regulates the expression of PD-L1 on antigen-presenting cells which affects the drug effect of immunotherapy (Wolfle et al., 2011).

### 3.5 Indirect effects on immune cells

A study found that STAT3-mediated IL-10 secretion can promote the formation of M2 macrophages, and M2 macrophages regulate the function of breast cancer stem cells through EGFR/STAT3/SOX-2 paracrine signals (Yang et al., 2013). IL-4 induces pSTAT6-mediated inhibition of the activation of multiple genes involved in alternative macrophage polarization, such as NLRP3 and IL-1B, thereby inhibiting inflammasome stimulation and pyroptosis (Czimmerer et al., 2018). In tumor-associated macrophages (TAMs), STAT1 regulates the expression of arginase and NO, which in turn suppresses T cell-mediated immune responses and induces T cell apoptosis (Kusmartsev and Gabrilovich, 2005; Alvaro et al., 2006). On the other hand, inactivated STAT3 in hematopoietic stem cells shows an anti-tumor effect, inhibiting tumor growth and metastasis by affecting the activation of DC, T, and NK cells (Kortylewski et al., 2005). The absence or targeting of STAT3 in myeloid cells can enhance CD8^+^ T cell responses and activate tumor-associated monocytes and DC cells, leading to anti-tumor responses (Herrmann et al., 2010), and inhibiting the pro-angiogenic factors VEGF, bEGF, MMP9, CXCL2 secretion, thereby inhibiting the formation of vascular-like structures (Kujawski et al., 2008). In addition, STAT1 is involved in the early development of B cells (Najjar et al., 2010).

## 4 Extracellular cytokines affecting the JAK/STAT pathway

**FIGURE 3:**
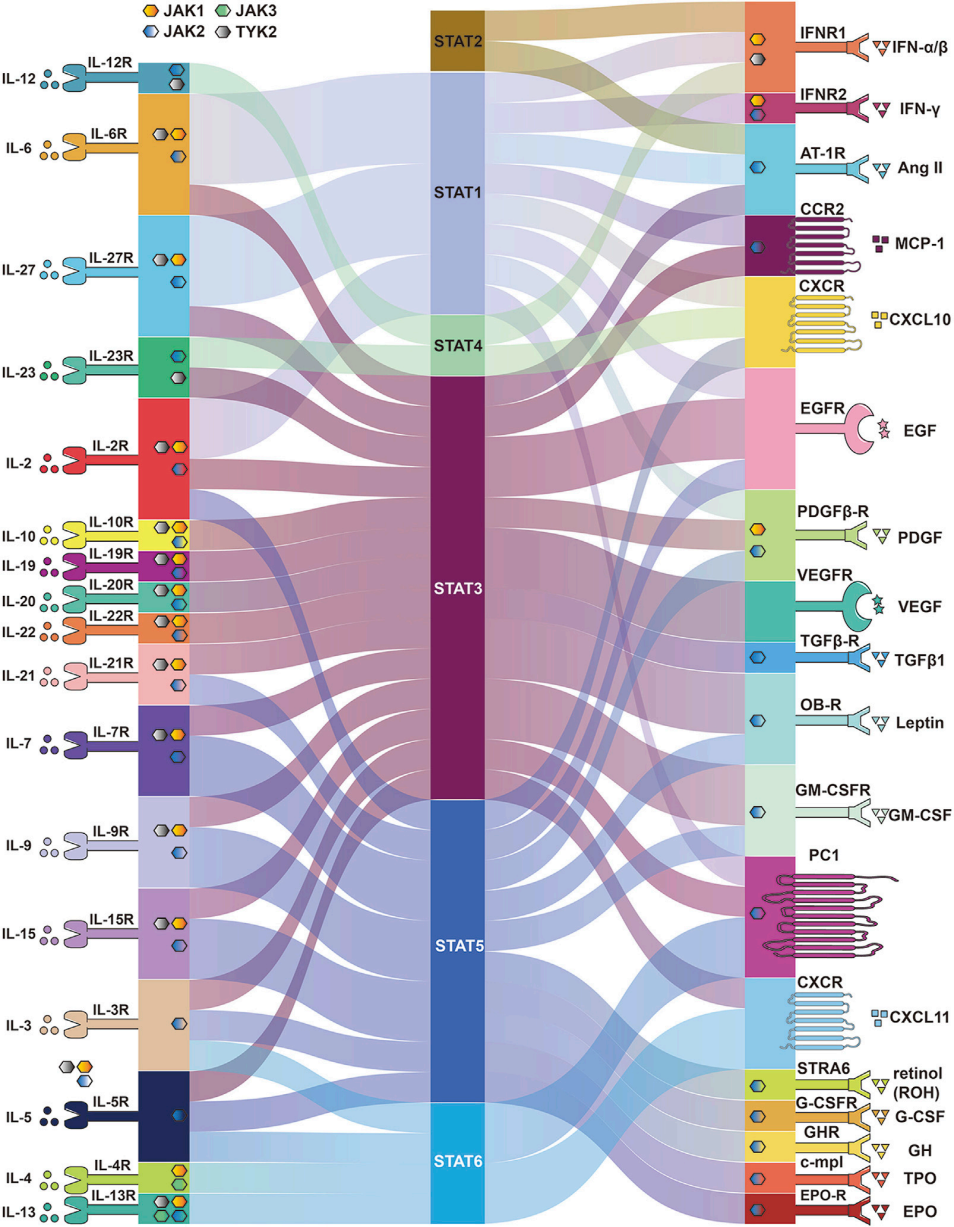
Membrane proteins and cytokines that activate the JAK/STAT pathway. Different membrane proteins and upstream cytokines phosphorylate different JAK and activate different STATA pathways. Some membrane proteins do not require JAK to activate STAT.

Many cytokines activate the JAK/STAT pathway, which transmits signals directly to the nucleus to induce various cellular responses (Figure 3). Below we describe the various cytokines and proteins that affect the JAK/STAT pathway and the various cellular activities to understand how the JAK/STAT pathway is involved in disease development and thus suggest more effective therapeutic approaches.

### 4.1 Interleukin in JAK/STAT pathway

Cytokines of the interleukin family are involved in multiple aspects of cellular life activity functions, including the immune system, physiological functions, inflammatory responses, and cellular metabolism, and all interferons activate members of JAK and STAT. It was shown that IL-6 activated gp130 leading to activation of the JAK/STAT pathway (Rose-John, 2018) and the IL-6/JAK/STAT3 pathway is aberrantly hyperactivated in many types of cancer (Johnson et al., 2018). Studies showed that IL-6 downregulated PTPRO expression leading to enhance PD-L1 secretion in monocytes and macrophages through the JAK2/STAT1 (Zhang W. et al., 2020). IL-4 and IL-13 activate JAK1 by binding to receptors, and JAK1 phosphorylates STAT6, thereby mediating their pulmonary fibrotic effects (Jakubzick et al., 2004), while IL-6 induced fibrosis by activating STAT3 (O'Donoghue et al., 2012). Chemokines trigger receptor dimerization, followed by association and activation of JAK proteins (Soriano et al., 2003). IL-12 is the main driver of STAT4 activation and crucial for IFN-γ production in NK cells (Wang et al., 1999). Studies showed that IL-23 induces IL-17A expression in macrophages through the STAT3 (Hou et al., 2018) and requires STAT4 for IL-17 secretion from memory T helper cells and NKT cells (Glosson-Byers et al., 2014). IL-10 and its subfamily member cytokines IL-19, IL-20, and IL-22 are involved in immune regulation and inflammatory responses by inducing the STAT3 signal transduction pathway (Conti et al., 2003). IL-2 family cytokines including IL-2, IL-4, IL-7, IL-9, IL-15, and IL-21 are involved in NK cell development by activating different JAKs and STATs (Gotthardt et al., 2019). IL-27 mediates signaling predominantly through STAT1 and STAT3 and acts in immune-regulatory functions (Fabbi et al., 2017).

### 4.2 Other cytokines in JAK/STAT pathway

Cytokines bind to receptor proteins on the cell membrane, activating the downstream JAK/STAT pathway or directly recruiting STAT protein without JAK involvement. Type 1 interferons IFN-α and -β signal bind to their membrane subunits IFNAR1 and IFNAR2 expressed on all cells, then triggering JAK1 and TYK2 phosphorylation and thus recruiting STAT1 and STAT2 monomers for their dimerization activation (Fuertes et al., 2013), thereby facilitating the initiation of dendritic cells (DC) required for T cell activation (Gessani et al., 2014), supporting immune cell migration, stimulation, and differentiation (Hervas-Stubbs et al., 2011), as well as inducing regulation of the PI3K/AKT/mTOR signaling pathway (Bai et al., 2009). Similar to type 1 interferon IFN, IFN-γ signals through two transmembrane receptor subunits, IFNR1 and IFNR2, activating receptor-associated JAK, leading to the selective recruitment of STAT1 (Braumuller et al., 2013), which stimulates the toxic function of CD8^+^ T cells and NK cells (Bhat et al., 2017; Takeda et al., 2017), as well as promoting macrophage polarization toward the M1 phenotype (Mills et al., 2000). Other studies in human fibroblasts showed that JAK2 has been activated by TGFβ1, then, in turn, phosphorylates STAT3 and leads to its nuclear translocation (Liu et al., 2013). Tumor-derived GM-CSF activated neutrophils and induced neutrophil PD-L1 expression via the JAK-STAT3 pathway (Wang et al., 2017). The effects of EPO, TPO, G-SCF, GH, and Leptin are mainly mediated by JAK2 and mainly STAT5 (Cirillo et al., 2008; Mullen and Gonzalez-Perez, 2016; Muller, 2019). Activation of angiotensin II (AT1) receptors has also been shown to phosphorylate STAT 1, 2, and 3 (Liang et al., 1999; Ram and Iyengar, 2001). Studies have demonstrated that STRA6, a plasma membrane protein, can act as a cell factor receptor that mediates the transport of retinol from serum RBP into cells to activate JAK2/STAT5 signaling (Berry et al., 2012). Studies showed that CCR2 tyrosine phosphorylation is associated with the JAK and STAT1/3 pathway at different stages of rat adjuvant-induced arthritis (AIA), as well as with macrophage and endothelial cell infiltration (Shahrara et al., 2003; Zhu et al., 2021). Studies indicated that JAK1 is a downstream tyrosine kinase in PDGF receptor signaling and is a candidate for activation of STAT1 (Choudhury et al., 1996). Furthermore, the expressions of PDGFRβ, JAK2, and STAT3 can be inhibited by AG490 (Jin et al., 2020). It was also found that *in vitro* PDGF-induced STAT5 activation was directly mediated by PDGFβ-R and its activation did not require JAK1, JAK2, c-Src, Fyn (Paukku et al., 2000). EGF-R is a transmembrane protein tyrosine kinase and EGF can direct activation of STATs by EGFR binding and indirect activation of STATs through Src-mediated EGFR signaling (Quesnelle et al., 2007). EGF induces activation of STAT1, STAT3, and STAT5 in a variety of EGFR overexpressing cells (Zhang et al., 2003). The study showed no phosphorylation of JAK kinase after the addition of VEGF, suggesting that STAT activation is induced by the intrinsic tyrosine kinase activity of VEGFR (Yahata et al., 2003; Roger et al., 2021). In conclusion, the JAK/STAT pathway is involved in multiple signaling cascades of life activities, and targeting these signaling may provide new ideas for disease treatment.

## 5 The effect of biophysical forces on the JAK/STAT pathway

\Cells can sense their extracellular environment and respond to chemical (Armstrong et al., 2017), optical (Pail et al., 2011), thermal (McGlynn et al., 2021), and biophysical forces (Pan et al., 1999; Ruwhof and van der Laarse, 2000) signals, which can affect downstream cellular signaling pathways via second messenger cascades (Pan et al., 1999). Ultimately, these changes alter cell behavior and functions (Komuro et al., 1990; Sadoshima and Izumo, 1993; Manokawinchoke et al., 2021). Unlike the effects of drug stimulation or gene editing on cellular activity and pharmacological responses (Bhardwaj et al., 2014), cells are often subjected to continuous and weak mechanical forces from the surroundings (Wang et al., 2010; Wang et al., 2016; Ding et al., 2017). Even various cell-ECM interactions (Guidolin et al., 2018) and 3D cell culture systems have specific biophysical forces (Bouet et al., 2015). These physical interactions continuously transmit extracellular signals to the cell nucleus, which have multiple and profound effects on numerous biological processes including membrane proteins (Dalton and Lemmon, 2021), intracellular organelles (Ma et al., 2019), nuclear transcription and translation processes (Graham and Burridge, 2016; Lee et al., 2021), and intracellular phase separation (Zhang J. Z. et al., 2020).

### 5.1 Biophysical forces in regulating the JAK/STAT pathway

**FIGURE 4:**
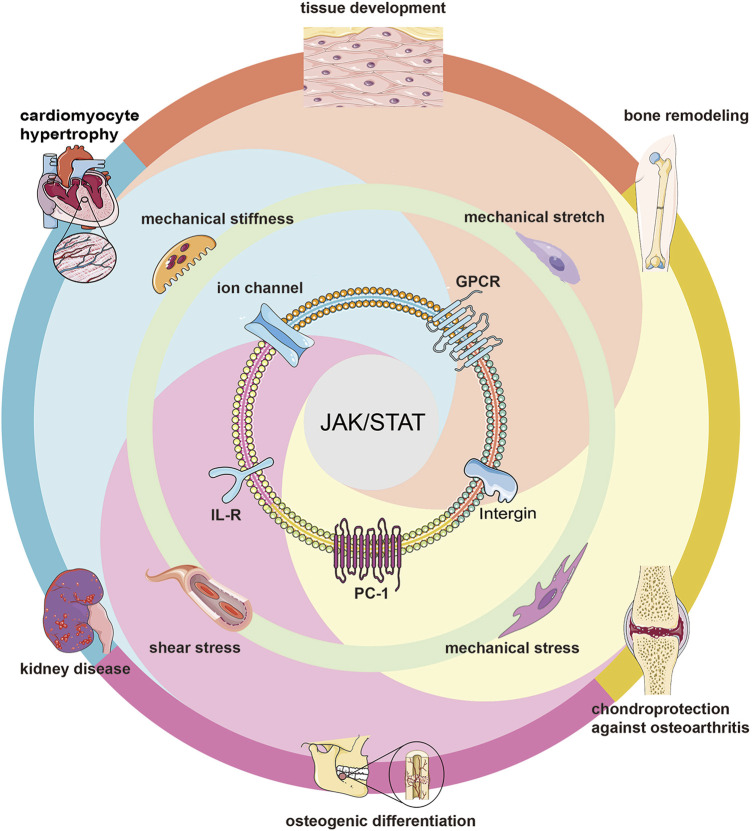
Mechanotransduction of JAK/STAT pathways. Different biophysical forces activate the JAK/STAT pathway through different membrane proteins, thus affecting many biological processes including tissue development, osteogenic differentiation, cardiomyocyte hypertrophy, etc.

The most common biophysical forces applied to mammalian cells are compression, stretching, shear stress, substrate stiffness, and substrate surface patterns (Wang et al., 2009b; Wang et al., 2009c; Wang et al., 2011; Wang et al., 2012; Wang and Tsai, 2013), which lead to morphological changes, cell membrane deformation (Manokawinchoke et al., 2021), membrane protein conformational changes (Ploscariu et al., 2014; Chen et al., 2017; Martinac and Poole, 2018), and ultimately triggering downstream signalings (Banes et al., 1995; Chen et al., 2017; Schneider et al., 2017). While cytoskeletons, ion channels, integrin receptors, G protein-coupled receptors, a transmembrane protein, and primary cilia are transit points for mechanical signals (Alfieri et al., 2019) and often influence the activation of downstream JAK/STAT pathways (Figure 4).

The study of biophysical forces in adult bone differentiation has been of interest, with several studies demonstrating that aged osteoblasts are characterized by impaired mechanosensitivity, and Cui et al. identified changes in the JAK/STAT pathway after transcription of the osteoblast transcriptome by transcriptomics (Cui et al., 2022). For the past few years, Jiliang Li has suggested that the JAK/STAT pathway plays an important role in bone development and metabolism, and that STAT3 has a more profound impact on bone homeostasis compared with other type of STATs (Li, 2013). Similarly, Natalie A Sims also held the same views and believed that the JAK1/STAT3/SOCS3 axis featured in bone development, physiology and pathology (Sims, 2020). Meanwhile, recent studies have shown that mechanical stimulation improves rotator cuff tendon-bone healing by activating IL-4/JAK/STAT signaling pathway mediated macrophage-M2 polarization (Liu et al., 2022). All in all, these studies indicate that JAK/STAT pathway plays an important role in bone development and repair.

Other types of biophysical forces such as extracellular matrix (ECM) stiffness, cell geometry, and shear stress were explored to activate JAK/STAT pathway signaling through activation of associated G proteins as well as rearrangement of the actin cytoskeleton (Erdogmus et al., 2019; Luo and Yu, 2019). Fong et al. found that mechanical shear stress can down-regulate PDGF (Fong et al., 2005) and thus modulate biological responses, a phenomenon that may be related to PDGF stimulation of primary cilia to induce STAT pathway activation. Static mechanical compressive forces lead to IL6 expression, and IL6 may subsequently indirectly activate STATs and translocate them to the nucleus through the JAK (Manokawinchoke et al., 2021). Also, it has been reported that cyclically stretch could induce the expression of MMP-14 and -2 in neonatal rat cardiomyocytes through JAK-STAT1 pathway (Wang T. L. et al., 2004). Braile and Jayaraman et al. found that pulsatile stretching can stimulate VEGF production in cardiomyocytes (CM) and that VEGF receptors can activate STAT phosphorylation, indirectly affecting downstream signaling via the JAK/STAT pathway (Seko et al., 1999; Jayaraman et al., 2012; Braile et al., 2020). In addition, Liang et al. also found that mechanical stretching-induced upregulation of VEGF-A in human mesenchymal cells was also associated with the JAK/STAT pathway, further illustrating the effect of biophysical forces on the JAK/STAT pathway (Liang et al., 2016).

### 5.2 JAK/STAT pathway mediates the role of biophysical forces

Current studies have shown that JAK/STAT-mediated mechanotransduction often has important effects on cell physiological processes. Matthews et al. found that mechanical stretch could affect the calcium influx by acting on β1 integrin (Matthews et al., 2010) and that Ca^2+^ plays a key role in stretch-induced activation of STATs (Pan et al., 1999), which has implications for cell and tissue development (Matthews et al., 2010). The research of Xiao et al. claimed that mechanical stretching-induced vascular endothelial growth factor A upregulation was related to the Janus kinase/signal transducer and activator of transcription (JAK/STAT) and Wnt signaling pathway (Liang et al., 2016). Researchers using mechanical stretching of human osteoblasts found that the stretching could upregulate Runx2 gene expression by enhancing the PC1-JAK2/STAT3 signaling axis, which has a decisive effect on bone remodeling (Dalagiorgou et al., 2017). Others showed that mechanical stress induced CCL2 (Zhu et al., 2021) to bind to CCR2 to regulate the production of osteoclasts in pressure grooves (Tsutsumi et al., 2013) and mediated chemotaxis and migration induction through activation of the JAK/STAT pathway *in vitro* and *in vivo* (Burysek et al., 2002). He et al. found that mechanical stress in chondrocytes combined with IL-4 to induce CITED2 gene expression in human chondrocytes via JAK/STAT pathway, thereby inhibiting matrix metalloproteinase (MMP13) production and providing chondroprotection against osteoarthritis (OA) (He et al., 2019). In addition, Qin et al. also found that periodontal ligament stem cells (PDLSC) sensitive to mechanical loading may downregulate HHIP-AS1 and promote the osteogenic differentiation potential of PDLSCs under continuous compressive stress, possibly via the JAK/STAT pathway (Qin et al., 2021). The Zyxin/Ajuba family of LIM proteins is a class of proteins that responds to biophysical forces (Jia et al., 2020), and Ajuba can play an important role in cell migration and epithelial morphogenesis by separating JAK1 from the interferon receptor and acting as a *bona fide* inhibitor of IFN/JAK1/STAT1 function (Sahai and Marshall, 2002; Jia et al., 2020). Machida et al. found that cyclic tensile strain induced the expression of ADAMTS4, ADAMTS5, and MMP13 in human chondrocytes via the underlying JAK/STAT pathway (Machida et al., 2017).

Mechanical stretch studies on rat cardiomyocytes by Pan et al. (Pan et al., 1997; Pan et al., 1999)found that mechanical stretch-induced cardiomyocyte hypertrophy (Kunisada et al., 1996; Kodama et al., 1997; Lammerding et al., 2004; Sims, 2020), which was largely dependent on cytokines of the IL-6 family, with activation of the JAK/STAT (mainly JAK1/STAT1, STAT3, partially binding to JAK2 and TYK2 (Sims, 2020)) pathway mediated by its receptor gp130 (Ruwhof and van der Laarse, 2000). Additionally, studies have shown that renal epithelial cells are subjected to flow-induced shear stress within the nephron and that kidney disease is affected by activation of the JAK/STAT pathway, as found by RNA sequencing (Kunnen et al., 2018). Honsho et al. found that pressure-mediated hypertrophy and mechanical stretch produced a low-level expression of IL-1β (subinflammatory), whereas JAK/STAT pathway-mediated production of IGF-1 could maintain its adaptive compensation for hypertrophy and inhibition of interstitial fibrosis (Honsho et al., 2009). Otherwise, in the neurodevelopmental process, Ciliary neurotrophic factor (CNTF) could directly stimulate JAK-STAT and RAS-MAPK cascaded reactions, and STAT3 signaling was considered as a potential component of neural response to stress stimuli (Peterson et al., 2000). More interestingly, it has been demonstrated that mechanical stress stimulates cellular immune response through JAK/STAT signaling pathway in *Drosophila* larvae (Tokusumi et al., 2018). In addition, other immunological studies have illustrated that the FTO/SOCS1/YTHDF1 regulatory axis was vital to the stiffness-controlled macrophage inflammatory response, including the culture environment of hydrogel with higher hardness could inhibit the expression of FTO gene through JAK-STAT and NF-κB signals (Hu et al., 2022).

In conclusion, biophysical forces occupy an important role in the induction of downstream signaling in the JAK/STAT pathway, playing a crucial role in the development of individual tissues, including bone, liver, heart, brain, nerves and immune regulation.

## 6 Clinical status of JAK/STAT pathway inhibitors

Based on the critical role of JAK/STAT in disease pathology and pharmacology, it is not surprising that inhibitors targeting JAK/STAT have been proposed to treat these diseases. Many inhibitors based on the JAK/STAT pathway have entered preclinical studies and clinical trials in a variety of diseases to evaluate their safety and clinical efficacy.

### 6.1 JAK/STAT inhibitors

At present, a variety of inhibitors targeting the JAK/STAT pathway have been used clinically, mainly for the treatment of rheumatoid arthritis, canine dermatitis, psoriasis, ulcerative colitis, myelofibrosis, polycythemia vera, and Primary thrombocytosis (Table 2). Ruxolitinib, a JAK inhibitor, has been identified by the FDA as a clinical treatment for Polycythemia, myelofibrosis, chronic graft-versus-host disease (cGVHD), and Atopic dermatitis by targeting JAK1 and JAK2. A number of clinical trial studies are also validating its efficacy and safety for the treatment of other diseases such as Chronic myelomonocytic leukemia (CMML) (Hunter et al., 2021), peripheral T-cell lymphoma (PTCL) (Moskowitz et al., 2021), lichen planus (Brumfiel et al., 2022), and COVID-2019 (Cao et al., 2020). The JAK inhibitor Tofacitinib is approved by the FDA as a treatment for rheumatoid arthritis, and it has also been used to study its effectiveness in Psoriasis (Abe et al., 2017), ulcerative colitis (Mukherjee et al., 2022), juvenile idiopathic arthritis (JIA) (Ruperto et al., 2021), transplant rejection (Vincenti et al., 2012), systemic sclerosis (SSc) (Khanna et al., 2022), Sarcoidosis (Damsky et al., 2022), systemic lupus erythematosus (SLE) (Hasni et al., 2021), and ankylosing spondylitis (AS) (Deodhar et al., 2021). Investigators are enrolling patients in a Phase IV clinical study of baricitinib for the treatment of Rheumatoid Arthritis, which is expected to become the standard of care for RA (NCT05238896). Oclacitinib is another inhibitor that targets JAK1 and is used to treat Canine allergic dermatitis (Little et al., 2015). Preclinical studies have shown that specific JAK2 inhibitors can inhibit the growth of tumors *in vivo*, including pancreatic cancer, colorectal cancer, gastric cancer, liver cancer, lung cancer, ovarian cancer, and breast cancer (Hedvat et al., 2009; Sun et al., 2011). Mohrherr et al. found that the JAK inhibitor Ruxolitinib reduced the proliferation of human K-ras-mutated A549 cells transplanted into immunodeficient mice, and decreased the expression of tumor cell-derived pro-cancer factors IL-1β and IL-6 (Mohrherr et al., 2019). And studies have shown that JAK2 inhibitor TG101209 can inhibit T cell acute lymphoblastic leukemia (T-ALL) proliferation by inhibiting JAK/STAT pathway activation and regulating the interaction between apoptosis and autophagy (Cheng et al., 2017).

**TABLE 2:** Clinical study of JAK/STAT inhibitors.

Agent	Target(s)	Disease(s)	Phase	Status*	ClinicalTrials.gov identifier(s)
Baricitinib	JAK1, JAK2	Rheumatoid Arthritis (RA)	Phase 4	Recruiting	NCT05238896
		Atopic Dermatitis	Phase 3	Completed	NCT03559270 NCT03435081
		Diabetic Nephropathy	Phase 2	Completed	NCT01683409
		Psoriasis	Phase 2	Completed	NCT01490632
		Alopecia Areata	Phase 2/3	Active, not recruiting	NCT03570749
		Systemic Lupus Erythematosus (SLE)	Phase 3	Completed	NCT03616964
		Pyoderma Gangrenosum	Phase 2	Recruiting	NCT04901325
		COVID-19	Phase 2/3	Completed	NCT04358614
		Human Immunodeficiency Virus	phase 2	Not yet recruiting	NCT05452564
		Dermatomyositis	Phase 3	Recruiting	NCT04972760
		Amyotrophic Lateral Sclerosis	Phase 1/2	Recruiting	NCT05189106
		Graft-versus-host-disease	Phase 1/2	Active, not recruiting	NCT04131738
		Systemic Sclerosis	Phase 4	Recruiting	NCT05300932
		Immune Thrombocytopenia	Phase 2	Recruiting	NCT05446831
		Juvenile Idiopathic Arthritis	Phase 3	Completed	NCT03773978
		Aicardi Goutieres Syndrome	Phase 2	Active, not recruiting	NCT03921554
		Liver Diseases	Phase 1/2	Completed	NCT01870388
		Arteritis	Phase 2	Completed	NCT03026504
		Sjogren’s Syndrome	Phase 2	Recruiting	NCT05016297
		Allergic Contact Dermatitis	Early Phase 1	Recruiting	NCT03945760
		Vitiligo	Phase 2	Active, not recruiting	NCT04822584
		Cutaneous Lichen Planus	Phase 2	Recruiting	NCT05188521
		Polymyalgia Rheumatic (PMR)	Phase 2	Recruiting	NCT04027101
		Idiopathic Inflammatory Myopathies	Phase 2	Recruiting	NCT04208464, NCT05400889
		Chronic Kidney Diseases	Phase 2	Recruiting	NCT05237388
Ruxolitinib	JAK1, JAK2	Polycythemia, Myelofibrosis, chronic Graft-versus-host disease (cGVHD), Atopic Dermatitis	FDA approved		
		Chronic Myelomonocytic Leukemia (CMML)	phase 2	Recruiting	NCT03722407
		Chronic Lymphocytic Leukemia	Phase 1/2	Completed	NCT02015208
		Lymphoma	Phase 2	Recruiting	NCT02974647, NCT01965119
		Lichen Planus	phase 2	Not yet recruiting	NCT05593432, NCT05593445
		Bronchiolitis Obliterans Syndrome	Phase 2	Recruiting	NCT05413356
		Vitiligo	Phase 3	Active, not recruiting	NCT04530344
		Chronic Hand Eczema (CHE)	Phase 3	Recruiting	NCT05233410
		COVID-19	Phase 2	Unknown	NCT04414098
		COVID-19 Induced Lung Injury ARDS	Phase 2	Completed	NCT04359290
		COVID-19 Associated Cytokine Storm	Phase 3	Completed	NCT04362137
		Thrombocythemia and Polycythemia Vera	Phase 2	Recruiting	NCT04644211
		Hemophagocytic Syndrome (HPS)	Phase 2	Completed	NCT02400463
		Solid Organ Transplant Recipients with Advanced Cutaneous Squamous Cell Carcinoma	Phase 2	Recruiting	NCT04807777
		Head and Neck Squamous Cell Carcinoma	Phase 2	Recruiting	NCT03153982
		Premalignant Breast Disease	Phase 2	Recruiting	NCT02928978
		Non-small Cell Lung Cancer cachexia	Early Phase 1	Recruiting	NCT04906746
Tofacitinib	JAK3, JAK1, JAK2	Rheumatoid Arthritis (RA)	FDA approved		
		Psoriasis	phase 2	Completed	NCT01831466
		Kidney Transplantation	Phase 2	Completed	NCT00263328
		Systemic Sclerosis (SSc)	phase 1/2	Completed	NCT03274076
		Sarcoidosis	phase 1	Completed	NCT03910543, NCT03793439
		Systemic Lupus Erythematosus (SLE)	Phase 2	Recruiting	NCT03288324
		Ankylosing Spondylitis (AS)	Phase 3	Completed	NCT03502616
		Ulcerative Colitis	Phase 3	Recruiting	NCT04624230
		Juvenile Idiopathic Arthritis (JIA)	phase 3	Completed	NCT02592434
		Alopecia Areata	Phase 4	Completed	NCT03800979
		Primary Sjögren’s Syndrome	Phase 2	Recruiting	NCT05087589
		Dermatomyositis	phase 1	Completed	NCT03002649
		Glioblastoma	Phase 2	Recruiting	NCT05326464
		Myasthenia Gravis	Early Phase 1	Recruiting	NCT04431895
		Psoriatic Arthritis	phase 3	Completed	NCT03736161, NCT03486457
		COVID-19	phase 2	Completed	NCT04750317
		Takayasu Arteritis	Phase 4	Recruiting	NCT05102448
Upadacitinib (ABT494)	JAK1	Rheumatoid Arthritis	Phase 3	Completed	NCT02955212
		Psoriatic Arthritis	Phase 3	Active, not recruiting	NCT03104374, NCT03104400
		Atopic Dermatitis	Phase 3	Active, not recruiting	NCT04195698, NCT03569293
		Hidradenitis Suppurativa (HS)	Phase 2	Completed	NCT04430855
		Spondyloarthritis	Phase 3	Active, not recruiting	NCT04169373
		Juvenile Idiopathic Arthritis (JIA)	Phase 1	Recruiting	NCT03725007
		Ulcerative Colitis (UC)	Phase 3	Completed	NCT03653026
		Crohn’s Dsease	Phase 3	Completed	NCT03345836, NCT03345849
		Takayasu Arteritis (TAK)	Phase 3	Recruiting	NCT04161898
		Ankylosing Spondylitis (AS)	Phase 2	Completed	NCT03178487
		Non-Segmental Vitiligo	Phase 2	Active, not recruiting	NCT04927975
		Giant Cell Arteritis (GCA)	Phase 3	Recruiting	NCT03725202
Itacitinib (INCB039110)	JAK1, JAK2	Plaque Psoriasis	Phase 2	Completed	NCT01634087
		Myelofibrosis	Phase 2	Completed	NCT01633372
		Non-Severe Hemophagocytosis Lymphohistiocytosis	Phase 2	Recruiting	NCT05063110
		Advanced Hepatocellular Carcinoma	Phase 1	Recruiting	NCT04358185
		Graft-versus-host-disease	Phase 2	Completed	NCT03846479
		Bronchiolitis Obliterans Syndrome	Phase 1/2	Active, not recruiting	NCT03978637
		Systemic Sclerosis	phase 2	Not yet recruiting	NCT04789850
		Metastatic Synovial Sarcoma	Phase 1	Recruiting	NCT03670069
		Rheumatoid Arthritis	Phase 2	Completed	NCT01626573
		Cytokine Release Syndrome	Phase 2	Recruiting	NCT04071366
Filgotinib	JAK1	Rheumatoid Arthritis (RA)	Phase 3	Active, not recruiting	NCT03025308
		Cutaneous lupus erythematosus (CLE)	Phase 2	Completed	NCT03134222
		Fistulizing Crohn’s Disease	Phase 2	Completed	NCT03077412
		Ulcerative Colitis	Phase 3	Completed	NCT02914522
		Ankylosing Spondylitis	Phase 2	Completed	NCT03117270
		Lupus Membranous Nephropathy (LMN)	Phase 2	Completed	NCT03285711
		Small Bowel Crohn’s Disease	Phase 2	Completed	NCT03046056
		Psoriatic Arthritis	Phase 2	Completed	NCT03101670
		Sjogren’s Syndrome	Phase 2	Completed	NCT03100942
Deucravacitinib	Tyk2	Psoriasis	Phase 3	Recruiting	NCT05478499, NCT04036435
		Psoriatic Arthriti	Phase 3	Recruiting	NCT04908202, NCT04908189
		Nail Psoriasis	Early Phase 1	Not yet recruiting	NCT05124080
		Plaque Psoriasis	Phase 3	Recruiting	NCT04772079
		Alopecia Areata	Phase 2	Not yet recruiting	NCT05556265
		Crohn Disease	Phase 2	Recruiting	NCT04877990
		Ulcerative Colitis	Phase 2	Active, not recruiting	NCT03934216
		Subacute Cutaneous Lupus Erythematosus (SCLE)	Phase 2	Recruiting	NCT04857034
Lestaurtinib (CEP-701)	JAK2	Myelofibrosis	Phase 2	Completed	NCT00494585
		Acute Myeloid Leukemia	Phase 2	Completed	NCT00079482
		Neuroblastoma	Phase 1	Completed	NCT00084422
		Polycythemia Vera	Phase 2	Completed	NCT00586651
		Chronic Beryllium Disease (CBD)	Phase 2	Completed	NCT00586651
		Psoriasis	Phase 2	Completed	NCT00236119
		Prostate Cancer	Phase 2	Completed	NCT00081601
Ritlecitinib	JAK3	Rheumatoid Arthritis (RA)	Phase 2	Completed	NCT04413617
		Alopecia Areata	Phase 2/3	Completed	NCT03732807
		Non-segmental Vitiligo	phase 3	Not yet recruiting	NCT05583526
		Cicatricial Alopecia	phase 2	Not yet recruiting	NCT05549934
Abrocitinib	JAK1	Atopic Dermatitis	Phase 3	Completed	NCT04345367
		Prurigo Nodularis	Phase 2	Completed	NCT05038982
		Food Allergy	Phase 1	Recruiting	NCT05069831
Brepocitinib	JAK1, Tyk2	Cicatricial Alopecia	Phase 2	Recruiting	NCT05076006
		Active Non-Infectious Non-Anterior Uveitis	Phase 2	Recruiting	NCT05523765
		Dermatomyositis	Phase 3	Recruiting	NCT05437263
Delgocitinib	JAK1, JAK2, JAK3, Tyk2	Atopic Dermatitis	Phase 2	Completed	NCT03725722
		Frontal Fibrosing Alopecia	Phase 2	Recruiting	NCT05332366
		Chronic Hand Eczema	Phase 3	Recruiting	NCT05355818
Danvatirsen (AZD9150)	STAT3	Advanced Colorectal Carcinoma	Phase 2	Active, not recruiting	NCT02983578
		Advanced Lung Non-Small Cell Carcinoma	Phase 2	Active, not recruiting	NCT03819465
		Metastatic Squamous Cell Carcinoma of the Head and Neck	Phase 1/2	Active, not recruiting	NCT02499328
Fedratinib	JAK2	Myeloproliferative Neoplasm	Phase 2	Recruiting	NCT05177211
		Myelofibrosis	Phase 3	Recruiting	NCT03952039
OPB-31121	STAT3	Advanced Solid Tumors	Phase 1	Completed	NCT00955812
		Hepatocellular Carcinoma	Phase 1/2	Completed	NCT01406574
OPB-51602	STAT3	Malignant Solid Tumour	Phase 1	Completed	NCT01423903, NCT01184807
Oclacitinib	JAK1	Canine Allergic Dermatitis	FDA approved		
Momelitinib	JAK1, JAK2	Anemic Myelofibrosis	Phase 3	Active, not recruiting	NCT04173494
Peficitinib	JAK1, JAK3	Rheumatoid Arthritis (RA)	Phase 3	Completed	NCT03660059, NCT02305849
Decernotinib (VX509)	JAK3	Rheumatoid Arthritis (RA)	Phase 2/3	Completed	NCT01830985
AZD1480	JAK1, JAK2	Myelofibrosis	Phase 1	Completed	NCT00910728
Gandotinib (LY2784544)	JAK2^V617F^	Myeloproliferative Neoplasms	Phase 2	Active, not recruiting	NCT01594723

Inhibitors targeting STAT3, Danvatirsen (Reilley et al., 2018; Nishina et al., 2022), OPB-31121 (Bendell et al., 2014; Oh et al., 2015), OPB-51602 (Ogura et al., 2015; Wong et al., 2015), are in clinical trials in a variety of solid tumors, including colorectal cancer, non-small cell lung cancer, liver cancer, head and neck cancer, etc. Although still in Phase 1 trials, their feasibility for treating tumors is being further demonstrated. In addition, several inhibitors targeting the JAK/STAT pathway are being tested in multi-stage clinical trials in a variety of diseases, such as Momelitinib (Mesa et al., 2022), Peficitinib (Tanaka et al., 2021), itacitinib (Naing et al., 2022), AZD1480 (Plimack et al., 2013), Fedratinib (Harrison et al., 2022b), Gandotinib (Berdeja et al., 2018), Filgotinib (Meng et al., 2022), Upadacitinib (Deodhar et al., 2022), Decernotinib (Genovese et al., 2016), lestatitinib (Brown et al., 2021), Decernotinib (Fleischmann et al., 2015), lestaurtinib (Mascarenhas et al., 2019), abrocitinib (Reich et al., 2022), ritlecitinib (Winnette et al., 2022), brepocitinib (Peeva et al., 2022), deucravacitinib (Mease et al., 2022), delgocitinib (Worm et al., 2022). The JAK/STAT pathway is activated in a variety of common solid tumors contributing to an aggressive phenotype (Roxburgh and McMillan, 2016). Studies also have shown that Ruxolitinib can regulate the expression of phosphorylated STAT1 in patients with STAT1 Gain-of-function mutations, thereby restoring the toxicity function of NK cells (Vargas-Hernandez et al., 2018). Silibinin is a direct STAT3 targeting agent, which can not only reduce therapy-associated nephrotoxicity, neurotoxicity and cardiotoxicity in preclinical models but also has the potential to reverse cancer cell drug resistance (Bosch-Barrera et al., 2017). STAT3 inhibitor WP1066 also inhibited Treg and increased T cell toxicity in patients with melanoma brain metastasis (Kong et al., 2009).

### 6.2 Combination therapy with JAK/STAT inhibitors

As we know, the clinical drug tolerance of some tumors is gradually emerging, so combined JAK/STAT inhibitor therapy may be a new treatment strategy (Table 3). JAK inhibitor (AZD1480) combined with EGFR inhibitor (cediranib) reduces tumor volume and microvascular density by reducing hypoxia and macrophage infiltration (de Groot et al., 2012). Sun’s results showed that TG101209 increased radiosensitivity by inducing apoptosis and decreasing cell proliferation and vascular density in lung cancer (Zhang et al., 2015). But in our experiments, we found that the JAK2 inhibitor, WP1066, combined with radiotherapy failed to reduce cell viability in gastric cell lines, which may be related to the cancer specificity. Matthew conducted a phase I/II trial to study the safety and efficacy of combining trastuzumab with ruxolitinib in patients with trastuzumab-resistant metastatic HER2^+^ breast cancer. However, the results did not observe an improvement in patient PFS (Kearney et al., 2021). And Sukhmani found that momelotinib in combination with erlotinib did not appear to enhance the benefit of patients with EGFR-mutated NSCLC (Padda et al., 2022). Robert evaluated the JAKA1 inhibitor itacitinib in combination with corticosteroids or placebo for the treatment of acute GVHD, and the observed improvement in ORR at day 28 in the combination group did not reach the prespecified significance level (Zeiser et al., 2022). Filgotinib was found to improve signs and symptoms of rheumatoid arthritis, improve physical function, inhibit radiographic progression, and be well tolerated by RA patients with an inadequate response to methotrexate (MTX) (Combe et al., 2021). Studies have shown that after combined treatment with the STAT3/5 inhibitor Static, the Oncolytic Adenovirus XVir-N-31 has increased viral replication and increased virus-induced death of bladder cancer cells (Hindupur et al., 2020). Furthermore, some clinical trials are ongoing. A phase II clinical trial investigated the efficacy and safety of adding the BCL-XL/BCL-2 inhibitor navitoclax in patients with myelofibrosis who progressed on ruxolitinib therapy or responded suboptimally to ruxolitinib monotherapy. The results demonstrated that patients achieved durable SVR35 (≥35% spleen volume reduction) and improved TSS50 (≥50% reduction in total symptom score) (Harrison et al., 2022a).

**TABLE 3:** JAK/STAT inhibitors are used in combination with other drugs.

Agent	Disease(s)	Phase	Status*	ClinicalTrials.gov identifier(s)
Ruxolitinib + Chidamide	Peripheral Blood Stem Cell Transplantation	Phase 2	Recruiting	NCT05088226
				NCT04582604
Ruxolitinib + Radiation and Temozolomide	Glioma	Phase 1	Active, not recruiting	NCT03514069
Ruxolitinib + Trastuzumab	Metastatic HER2 Positive Breast Cancer	Phase 1/2	Completed	NCT02066532
Itacitinib + Everolimus	Classical Hodgkin Lymphoma	Phase 1/2	Recruiting	NCT03697408
Itacitinib + Low-Dose Ruxolitinib	Myeloproliferative Neoplasms (MPN)	Phase 2	Completed	NCT03144687
Itacitinib + Osimertinib	Non-Small Cell Lung Cancer	Phase 1/2	Active, not recruiting	NCT02917993
Itacitinib + Alemtuzumab	T-Cell Prolymphocytic Leukemia	Phase 1	Recruiting	NCT03989466
Itacitinib + Ibrutinib	Diffuse Large B-Cell Lymphoma	Phase 1/2	Completed	NCT02760485
Itacitinib + Corticosteroids	Acute Graft-versus-host disease	Phase 3	Completed	NCT03139604
Itacitinib + Gemcitabine and Nab-Paclitaxel	Pancreatic Cancer	Phase 1/2	Completed	NCT01858883
Itacitinib + Dabrafenib and Trametinib	Melanoma	Phase 1	Active, not recruiting	NCT03272464
Itacitinib + Pembrolizumab	Colorectal Cancer	Phase 1	Completed	NCT02646748
Fedratinib + Decitabine	Myeloproliferative Neoplasms (MPN)	Phase 1	Recruiting	NCT05524857
Fedratinib + Nivolumab	Myelofibrosis	Phase 2	Recruiting	NCT05393674
Decitabine + Ruxolitinib or Fedratinib	Accelerated/Blast Phase Myeloproliferative Neoplasms	Phase 2	Recruiting	NCT04282187
Filgotinib + Methotrexate	Rheumatoid Arthritis	Phase 3	Completed	NCT02886728
				NCT02889796
Upadacitinib + Methotrexate	Rheumatoid Arthritis	Phase 3	Recruiting	NCT05121298
Upadacitinib + Corticosteroids	Atopic Dermatitis	Phase 3	Completed	NCT03661138
Upadacitinib + Elsubrutinib	Systemic Lupus Erythematosus (SLE)	Phase 2	Completed	NCT03978520
Lestaurtinib + Chemotherapy	Acute Lymphoblastic Leukemia	Phase 3	Active, not recruiting	NCT00557193
Upadacitinib + Methotrexate	Rheumatoid Arthritis	Phase 2	Completed	NCT01960855
Danvatirsen + Tremelimumab	Diffuse large B-cell lymphoma	Phase 1	Completed	NCT02549651

Moreover, potential natural products such as plant-derived cucurbitacin I and curcumin analogue ASC-J9 could be used as JAK/STAT inhibitors to treat related diseases (Yin et al., 2023), and the small molecule drugs, which screened from the databases (Dogra et al., 2023), targeting JAK/STAT could also be regarded as treatment strategies for related diseases (various organ fibrosis, etc.) (Liu et al., 2023).

## 7 Conclusion

The JAK/STAT pathway, a key pathway for protein signaling on the membrane, is crucial in human cells. The dysregulation of this pathway has been considered one of the causes leading to disease progression and tumor growth. In tumors, JAK/STAT acts as a regulatory hub for transduction signals, which affects the activation of various inflammatory factors, growth factors, and angiogenic factors in the tumor microenvironment (TME), and participates in regulating the maturation, proliferation, and differentiation of various immune cells. In addition, JAK/STAT pathway is also affected by many extracellular mechanical signals and consequently mediates numerous downstream biological processes. Therefore, inhibition of this pathway has attracted widespread attention as a potential therapeutic strategy. Based on the underlying molecular and genomic mechanisms of JAK/STAT, the internal and external factors affecting JAK/STAT activity, epigenetic and transcription factors, and genetic causes of dysregulated JAK/STAT signaling, these provide good ideas for the development and implementation of targeted drugs. In order to properly incorporate JAK/STAT targeted drugs into multimodality therapies, including combinations with chemotherapy, radiotherapy, immunotherapy, and physiotherapy, we also need to find predictive biomarkers, not just the overactivation of pathways. In view of the different sensitivity of individuals to drugs, it will be a hot research direction for us to learn more about the changes in individual tumor genomes and help us make therapeutic regimens for different gene mutations in the future.
